# Modular titanium alloy neck failure in total hip replacement: analysis of a relapse case

**DOI:** 10.1051/sicotj/2016009

**Published:** 2016-04-29

**Authors:** Marco Ceretti, Francesco Falez

**Affiliations:** 1 Saint Filippo and Nicola Hospital Via Adolfo Omodeo 31 d CAP 00179 Rome, Avezzano Italy; 2 Saint Spirit Hospital CAP 00193 Rome Italy

**Keywords:** Modular neck, Prosthesis, Fatigue fracture, Total hip arthroplasty

## Abstract

Modular neck hip prosthesis born in the 1990 with the aim of allowing the surgeon to modify CCD angle, offset and femoral anteversion intra-operatively restoring patient’s original biomechanics. In order to achieve the best biomechanics of the reconstructed hip, preoperative planning is essential. In the last few years modularity has been questioned and an argument made for the return to mono block stems due to events of breakage or disconnection of modular components. Fretting or crevice corrosion may lead to failure of such modular device due to the contamination inside the modular coupling or to high loads. We present a case of repetitive modular femoral neck prosthesis fracture.

## Introduction

Modularity introduction in the last 1990 has changed the approach strategies in primary and revision total hip replacement. Neck modularity guarantees in primary total hip replacement more options to reproduce the physiological longitudinal and lateral offset and anteversion or retroversion. In total hip revision surgery, modularity has the most important role to adapt the old femoral implant to the new acetabular implant varying offsets with also the stability benefit of the overall system. Fracture of the neck is a risk in neck modularity, which many authors and those in industry are trying to resolve.

## Case report

A 43-year-old woman (BMI 38.6 kg/m^2^, weight 110 kg, height 170  cm) came to our observation in October 2006 with right hip pain. Clinical findings and radiograph imaging revealed right hip arthrosis and a varus short neck.

In November 2006, the patient underwent right total hip arthroplasty (THA), which was performed with the Hardinge lateral approach. The implant chosen was a modular uncemented Metha titanium stem (Aesculap Orthopaedics) with a 135° neck long, a 36 mm medium ceramic head (Biolox delta), a 50 mm Delta PF cup (Lima Corporate), and Liner in ceramic (Biolox delta). We also put a cerclage for a lesser crack at the little trochanter level.

The patient’s initial postoperative course was uncomplicated. After six months, HHS was 98 points and ROM was: flexion 110°, abduction 45°, extension 10°, internal rotation 15°, external rotation 15°.

After two years and five months, the patient came back with atraumatic right hip pain described as unremitting and was associated with grinding and clicking sensation during ambulation with an instability feeling.

**Figure 1.**
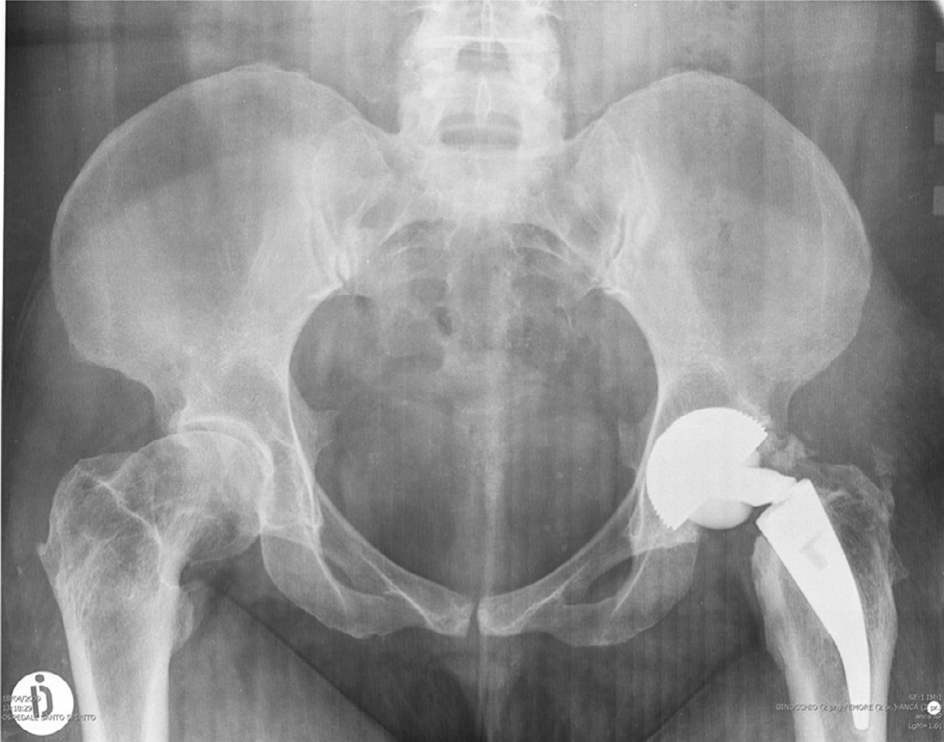
Neck-stem junction breaking in the absence of traumatic injury after two years from the 1st. The prosthesis is a Metha stem implant with modular neck. It was implanted in 2006 when the corrosion theory was not known and the modular neck was in titanium without CrCo.

X-rays demonstrated deformity at the neck-stem junction with increased neck varus angulation suggestive of a femoral neck fatigue fracture (Figure 1).

**Figure 2.**
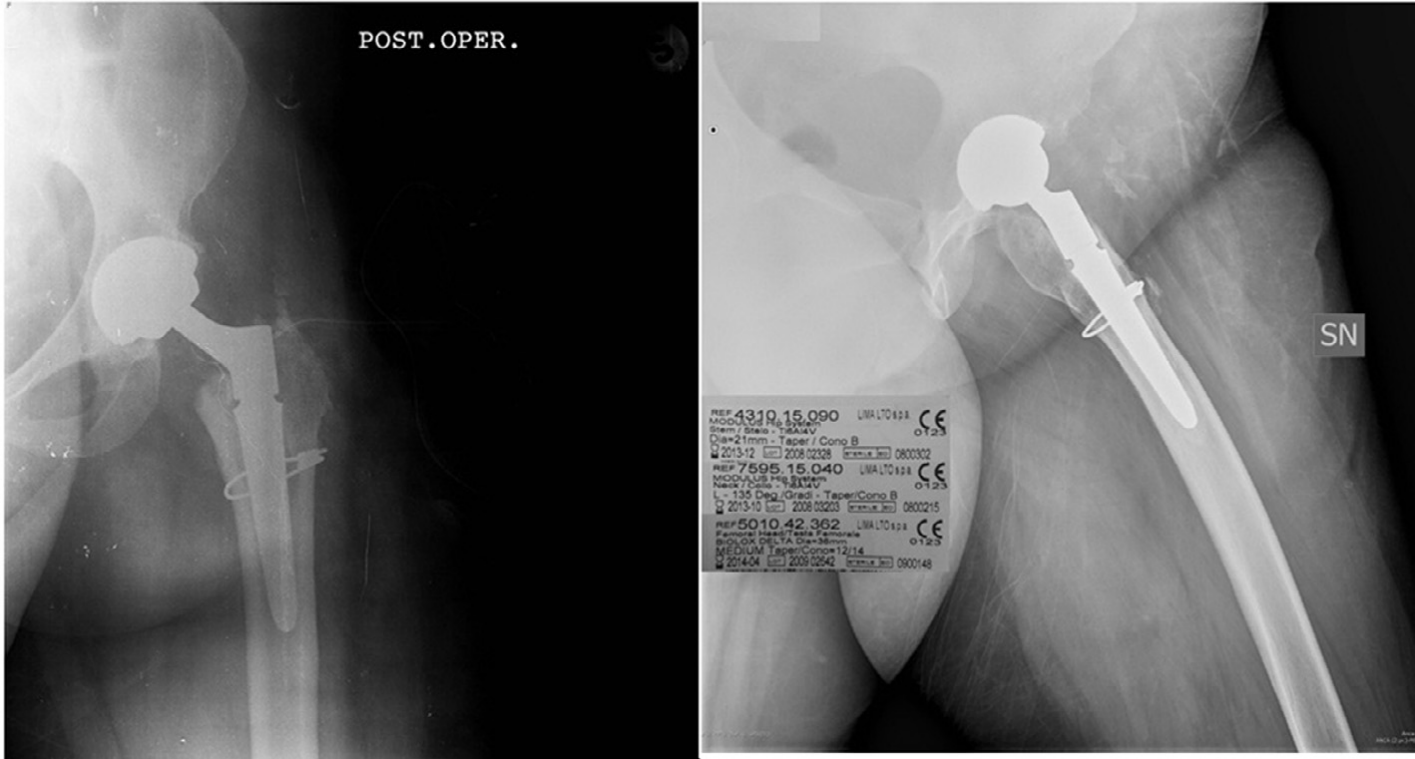
Postoperative X-ray after the Metha stem revision. The 1st implant revision was performed with Lima Modulus implant. Implant details: modulus stem 21 mm diameter, neck Ti6Al4V 135° taper B, Femoral Head 36 mm Biolox delta.

The patient was taken to the operating room for revision surgery. During the procedure it was possible to remove a part of the fractured femoral neck from the hip stem. We removed all the femoral implant via the Hardinge approach and it was decided to implant a modular uncemented Modulus titanium hip stem (Lima Corporate, diameter 21 mm taper B) with titanium long 135° neck and a 36 mm medium ceramic femoral head (Figure 2).

The patient’s postoperative course was uncomplicated.

**Figure 3.**
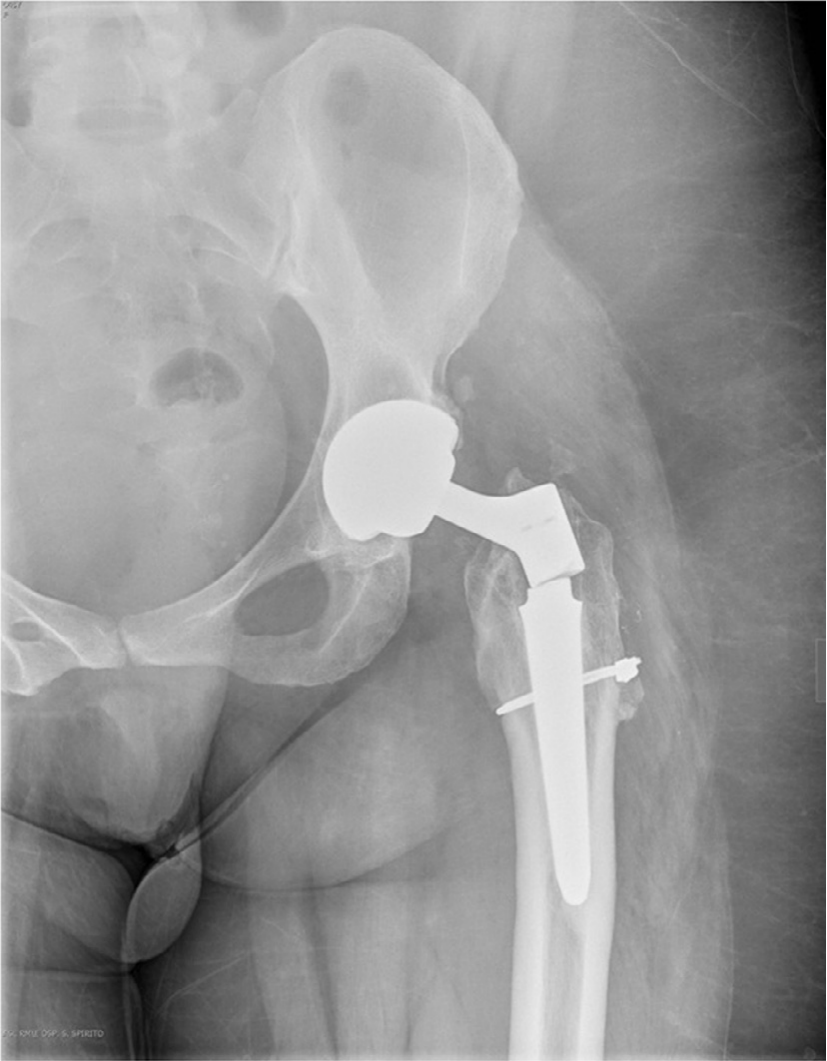
Neck-stem junction breaking in 1st revision implant after atraumatic pain two years after the revision. The rupture is localized on the thread at the neck-stem junction. The neck was virus (135°) in Ti6Al4V.

After six months, HHS was 88 points and ROM was: flexion 110°, abduction 40°, extension 10°, internal rotation 15°, external rotation 15°. After two years and six months, the patient felt the same non-traumatic right hip pain. X-rays demonstrated the stem rupture at the neck-stem junction (Figure 3).

**Figure 4.**
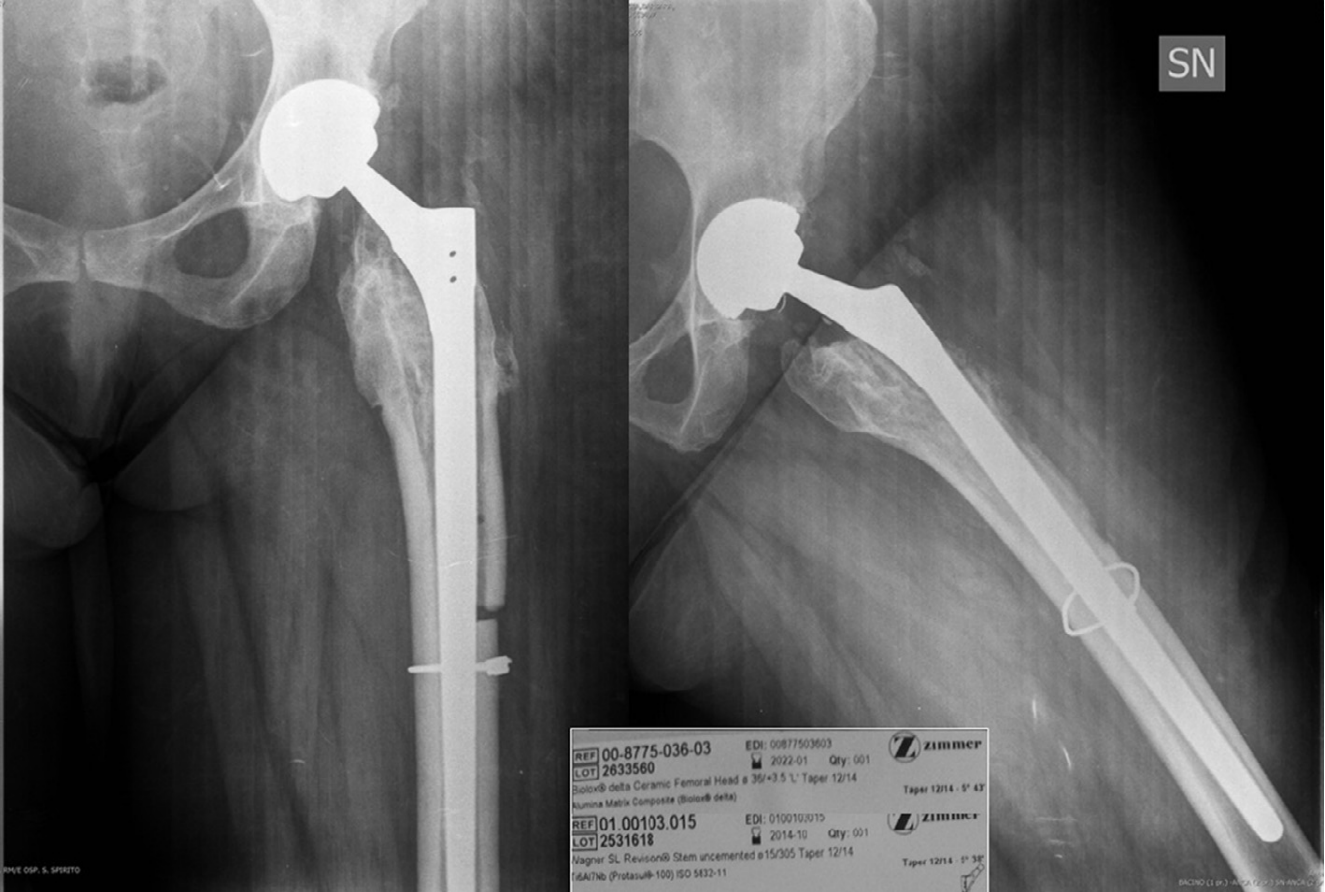
Postoperative X-ray after 2nd revision. The stem choice was a monolithic Wagner revision implant with the following details: femoral head 36 + 3.5, Lima Wagner Sl revision stem. The Wagner stem is already hole.

Our strategy was to remove the modulus hip stem using a Wagner osteotomy and our choice was to implant a Wagner monoblock uncemented titanium stem (Wagner SL revision produced by Zimmer) with a 36 mm + 3.5 Biolox delta ceramic head (Figure 4).

## Discussion

Modular hip systems offer the surgeon the potential to restore normal hip biomechanics with the ability to independently adjust offset, version, and limb length.

Fracture of the femoral component is a rare complication in total hip arthroplasty. Before the introduction of cobalt chromium molybdenum and titanium alloys, Charnley estimated the stem fracture prevalence to be 0.23% with other designs.

Modular titanium alloy neck fracture in Metha prosthesis is 1.4% and the higher incidence of failure is between nine and 42 months postoperatively. There is also a direct proportionality between patient weight, caput-collum-diaphysis (CCD) angle, and failure incidence [1].

In vitro studies of femoral components with neck-stem modularity have shown that corrosion and fretting can occur at the neck-stem junction [2, 3]. Corrosion and fretting occur at both the head-neck and the neck-stem modular junctions [4] and neck-stem junction degradation is more significant than at the head-neck junction and is believed to be secondary to the increased lever arm and high mechanical stress [2, 3]. Varus neck with increased offset and reduced length is the modular neck style, which creates the highest strain at the modular neck-stem junction and is similar to the geometry reported in other case reports of modular neck failure [5].

Specifically the use of a long varus neck increases the bending moment by 32.7% compared with the standard short varus neck with increasing stress concentration at the modular junction [6]. Crevice and fretting corrosion are higher in titanium alloy adapters compared with cobalt chrome [1].

Most reported fatigue fractures of modular stems have been associated with titanium (Ti6Al4V) alloy neck and stem components [1].

Micro-motions at the junctional interface induce fretting and crevice corrosion, contributing to micro-crack creation within the zone of corrosion and increasing the risk of dynamic fatigue fracture.

With the increased stresses on the modular head-neck and neck-stem junctions, oxide layers are disrupted, leading to a vicious cycle with the recurrent attempts of the metal to repassivate their surface, subsequently depleting available oxygen supply releasing chloride ions within the joint and lowering local pH.

This creates an anaerobic and acidic environment more conducive to further abrasive wear and corrosion, potentiating the risk of component fracture over time [1, 7, 8]. The combined effects of corrosion, large femoral head components with long modular necks, metal-on-metal components, patient obesity, and activity level may create a local microenvironment that can initiate and perpetuate fatigue failure. Intra-operative contamination of the cone connection with bone particles has a considerable impact on the magnitude of fretting in the interface due to the increased micro-motions [9].

In conclusion, we have formed the opinion that in the presence of a high BMI, a CCD angle < 135°, and a high functional demand the risk of failure is significantly increased in modular prostheses. We also believe that the modularity is not to be discouraged in an absolute manner, rather, if one chooses to go this route in such patients the preferred neck should be CoCrMo rather than Ti6Al4V.

## Conflict of interest

Dr. Ceretti does not have relevant financial relationships to disclose.

Dr. Falez is a paid consultant for Smith & Nephew, Lima, Samo, and DePuy; receives payment for lectures from Smith & Nephew, Lima, and DePuy; receives royalties from Smith & Nephew and receives payment for the development of educational presentations from Smith & Nephew, Lima, and DePuy.

The authors declare that there is no conflict of interest regarding the publication of this manuscript.
